# Chancre of the eyelid as manifestation of primary syphilis, and precocious chorioretinitis and uveitis in an HIV-infected patient: a case report

**DOI:** 10.1186/1471-2334-12-226

**Published:** 2012-09-25

**Authors:** Salvatore Cillino, Francesco Di Pace, Marcello Trizzino, Valentina Li Vecchi, Paola Di Carlo

**Affiliations:** 1 Department of Experimental Biomedicine and Clinical Neuroscience, Ophthalmology Section, University of Palermo, Via Liborio Giuffrè 13, 90127, Palermo, Italy; 2 Department of Sciences for Health Promotion “G. D’Alessandro”, Infectious Diseases Section, University of Palermo, Palermo, Italy; 3 Department of Internal Medicine and Specialties, University of Palermo, Palermo, Italy

**Keywords:** Ocular syphilis, HIV-infected, Chancre of the eyelid, Bilateral chorioretinitis, Uveitis

## Abstract

**Background:**

Ocular syphilis is often difficult to diagnose because of the wide variation in clinical features.

HIV co-infection may further complicate the picture.

**Case presentation:**

Herein the authors report an unusual primary syphilitic ocular lesion in a 45-year-old Italian HIV-infected bisexual man who presented with a unilateral eyelid lesion. Associated precocious signs and symptoms in the posterior segment of both eyes, bilateral chorioretinitis and uveitis, are described. Intravenous penicillin and steroid treatment produced a rapid improvement in clinical status and complete resolution.

**Conclusions:**

Careful questioning about sexual behavior is crucial for unmasking unusual features of ocular syphilis in HIV-infected subjects.

## Background

Recent reports show that ocular syphilis is becoming an increasingly common clinical problem, reflecting a growing incidence of syphilis in immunocompetent subjects of all ages and in particular in patients with HIV infection [1,2]. Eye infection can occur at any stage of the disease, and includes interstitial keratitis, anterior, intermediate, and posterior uveitis, chorioretinitis, retinitis, retinal vasculitis and cranial nerve and optic neuropathies [1-3]. Ocular findings may be associated with Central Nervous System involvement or be the sole presenting manifestation [3-6].

Attention has been mainly focused on *Treponema Pallidum* and HIV co-infection, as concurrent HIV frequently alters the natural course and typical clinical features of disease, and of neurosyphilis in particular [5,6]. Furthermore, clinical and laboratory diagnosis is often uncertain in HIV-infected patients [7]. Although specific treatment can improve vision, recurrences have been observed [2,5,7].

We report the favorable outcome of a case of bilateral chorioretinitis and uveitis in an HIV–infected patient, which started out as a unilateral ulcer on the lower eyelid.

### Case presentation

In January 2011, a 45-year-old Italian bisexual man, known to be HIV-infected since 1998, presented to the Infectious Diseases Unit with partial vision loss in both eyes.

Since 2000, HIV infection had been treated with combination antiretroviral therapy (cART) including zidovudine, lamivudine and lopinavir/ritonavir. Nadir absolute CD4+ T-cell count was 320/mm^3^.

The patient had been regularly attending different Day Hospital Services and was adhering well to cART. He had received two lines of cART, including ritonavir-boosted protease inhibitor regimens without experiencing virological failure.

On admission, his absolute CD4+ T-cell count was 385/mm^3^, HIV viremia was undetectable and his cART consisted of tenofovir emtricitabine and boosted atazanavir**.**

The patient's past treatment history revealed that he had received a single intramuscular injection of 2.4 million units of penicillin G benzathine for primary syphilis contracted in November 2000 after unprotected heterosexual exposure. At that time, he had a penile lesion with inguinal adenopathy, and he tested positive for syphilis as follows: serum Venereal Disease Research Laboratory (VDRL) test ++, *Treponema pallidum* haemoagglutination assay (TPHA) 1: 1280, positive FTA-Abs. Serological post-treatment follow-up showed that the VDRL test had reverted to non-reactivity within 12 months. His last known non-reactive VDRL test result was in June 2009.

Two weeks before admission to the Infectious Diseases Unit, the patient had noticed an ulcer on his left upper eyelid and four days before admission he had begun to experience reduced visual acuity, with associated photophobia and mild headache which had subsequently improved. His general practitioner suspected a chalazion and the patient was referred to our Ophthalmology Section because of visual impairment.

**Figure 1:**
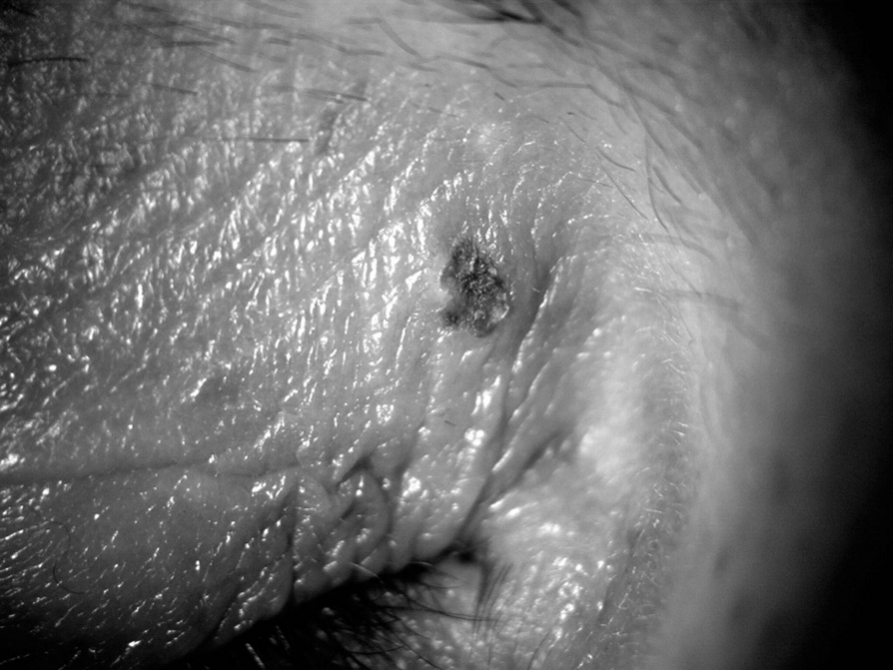
Ulceration above external canthus of the left eye.

A small, painless, resolving ulceration with barely elevated edges was present above the external canthus of the LE (Figure 1). No other abnormalities were seen in the remainder of his eyelids, eyelashes, cornea, bulbar conjunctiva, the other eye or on the rest of his face. His left preauricular and submandibular nodes were slightly enlarged, non-tender and firm.

**Figure 2:**
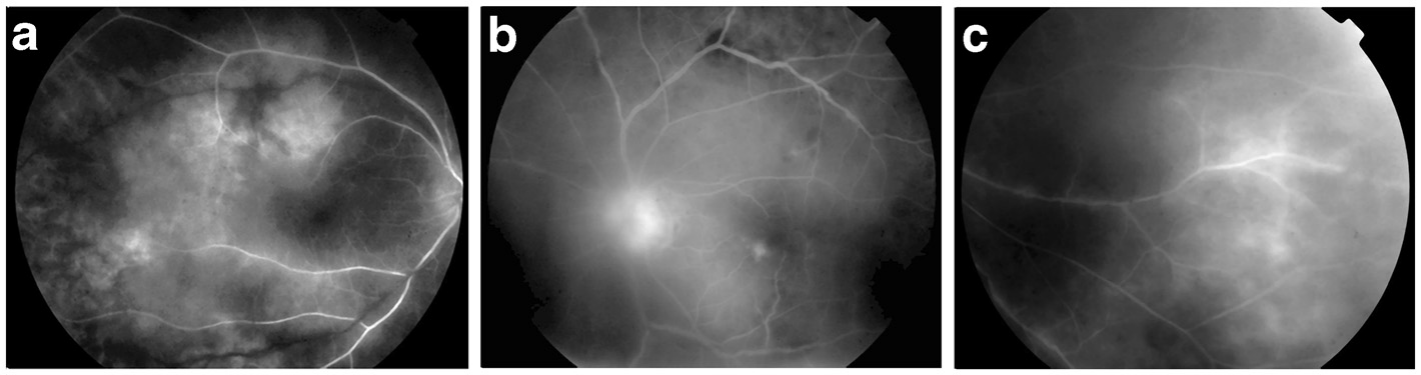
(2a,2b,2c) Fluorescein angiography with masked retinal areas and leakage.

Best corrected Snellen visual acuity was 20/32 in both eyes. Fine pigmented keratic precipitates, aqueous cells, flare and posterior synechiae were seen in the anterior chamber. Examination of the posterior chamber revealed vitreitis, retinal vasculitis with perivascular sheathing and hemorrhages, edematous retinal areas and disc swelling. Intravenous fluorescein angiogram showed masked retinal areas, optic disc hyperfluorescence and leakage from retinal venules (Figures 2a, 2b and 2c).

Structured face-to-face interviews were conducted with the patient to obtain information about his behavioral risk factors and symptoms. He had a history of multiple sexual partners in the previous year and unprotected oral sex intercourse with a sperm contamination of his eyes**.** His sexual contacts were traced and two immunocompetent young men tested positive for syphilis.

Complete blood count was unremarkable. Hematologic values were within normal range and Polymerase Chain Reaction (PCR) for *Toxoplasma gondii*, *Herpes viruses*, *Mycobacterium tuberculosis* (MT)*,* non-tuberculous mycobacteria (NTM) and other pathogens which are epidemiologically relevant in our geographic area (i.e. *Rickettsia conorii, Brucella spp*) gave negative results. Negative results were obtained for CMV DNA (using Real Time for amplification of the IE region) and CMV pp65 antigen in 200000 leukocytes. EBV-DNA (amplifying the Bam Hi W region) and HSV DNA (amplifying viral polymerases) were also negative. Serum VDRL titer was 1 : 16, and TPHA titer was 1 : 1280.

Macroscopic and humoral studies of cerebrospinal fluid (CSF) performed at the time of admission were negative. CSF VDRL and CSF PCR test results for the above-mentioned microorganisms were also negative.

The patient was treated with intravenous penicillin G (24 MU/day) for two weeks and received 1 intramuscular dose of penicillin G benzathine (2.4 MU) after completing the I.V. therapy [7,8].

He was treated topically with dexamethasone qid and atropine 1% bid, and was prescribed 60 mg of oral prednisone daily, tapered over 4 weeks.

At the two-week follow-up visit, papillitis had disappeared, and retinal vasculitis and uveitis had improved. Best corrected Snellen visual acuity remained unchanged. The topical therapy was tapered.

After one month, aqueous cells and flare had further decreased but some retinal hemorrhages and perivascular sheathing were still observed.

**Figure 3:**
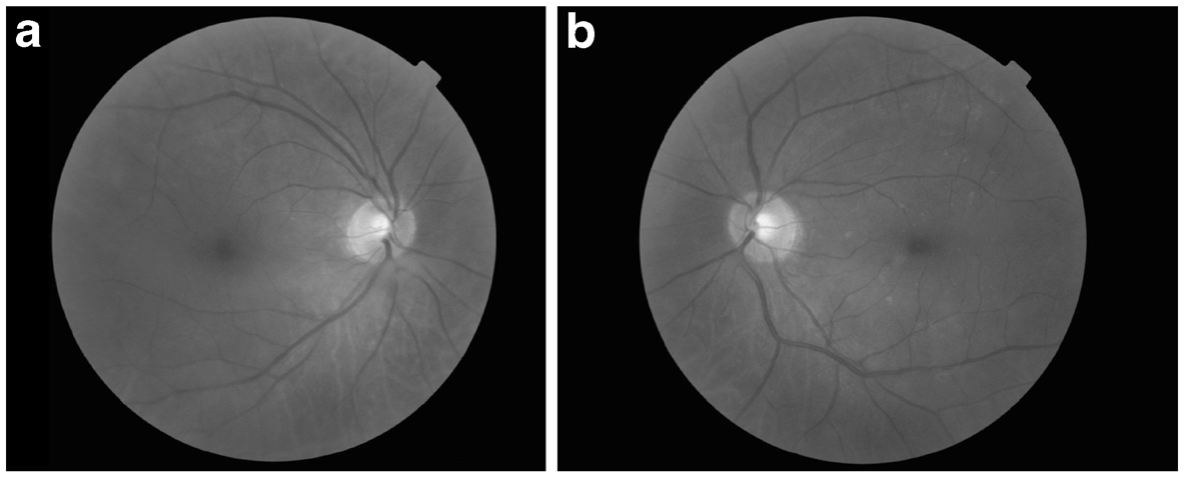
(3a,3b) Red-free fundus images of RE and LE at 2-months follow-up.

At 2-months follow-up, signs of uveitis had disappeared, posterior synechiae were sporadic and fundus examination showed a normal disc and peripheral hyalinized vessels with attached retina (Figures 3a and 3b).

One year after treatment, serological testing for syphilis was repeated, showing positive VDRL 1:2 and positive TPHA 1:80.

At present, the patient is regularly attending our Day Care Unit where we can monitor his adherence to therapy.

His last CD4+ T-cell count was 530cells/mm^3^ and viral load was undetectable.

Ocular examination showed that both eyes were quiet.

## Discussion

Due to changes in sexual behavior, a new approach to clinically evaluating sexually-transmitted diseases (STDs) is needed in order to detect primary and secondary findings in sites which are not usually investigated. Our report has shown that an external ocular lesion, involving the eyelid for example, may be a manifestation of primary syphilis. The lesion is typically a single chancre (a firm, painless, non-itchy skin ulceration) [9]. As we reported in case presentation, the lesion had been mistaken for a chalazion, but other infiltrative/ulcerative skin lesions in the eyelid, such as neoplasms, can be mimicked, so that the patient is referred to an ophthalmologist when the lesion involves the internal coats of the eye and the optic nerve. For instance, a chalazion can evolve into a pustule, which is a small elevation of the skin containing cloudy or purulent material usually consisting of necrotic inflammatory cells. The pustule can in turn be followed by an ulceration, vaguely resembling that of a primary syphilis chancre. Nevertheless, a chalazion is usually painful and lacking punched-out base and rolled edges.

To our knowledge, this is the first documented case of chancre of the eyelid in an HIV-infected patient. Jeyakumar described the same primary syphilitic manifestation in an immunocompetent patient, and in this case the lesion healed completely with a single deep intramuscular injection of benzathine penicillin 2.4 MU [9].

Until recently, in many clinical settings where dark-field microscopy was unavailable, a definite diagnosis of primary syphilis was based solely on clinical criteria and serologic testing. Now, *T. pallidum* real-time PCR is considered to be a fast and reliable test to detect primary syphilis [10].

In our patient’s case, we can consider the diagnosis of primary syphilis confirmed as it was based on several markers such as clinical features, serology, additional information on the patient's history, follow-up data after specific treatment, exclusion of other infectious diseases and the development of secondary manifestations. As suggested by Heymans, *T. pallidum* real-time PCR is useful for diagnosing primary syphilis in an STD outpatient clinic and in a general practitioner setting, but it has no added diagnostic value for the diagnosis of secondary syphilis [11].

We observed uveitis/retinitis in our patient fifteen days after the appearance of the primary eyelid lesion, and we suspected a precocious involvement of internal ocular coats. Secondary syphilis occurs approximately four to ten weeks after the primary infection [12]. Previous reports have suggested that uveitis occurs mainly in secondary or tertiary syphilis [3,13,14].

This case suggests that it may take a shorter time for secondary eye lesions to develop in the HIV population, especially in individuals who practice unprotected oral sex. Direct contamination of the eye with genital secretions containing treponemes may influence the evolution of syphilitic lesions in an immunologically privileged site such as the eye [15].

The rapid effect of specific anti-syphilis therapy with evidence of an improvement in ocular lesions is the most important factor for early diagnosis and complete recovery of the eye. There are reports on the adjunctive use of steroids to treat ocular syphilis [7]. In our patient’s case, systemic antibiotic treatment and topical and oral steroids led to substantial initial clinical improvement and a favorable outcome.

The extent of ocular involvement is unrelated to CD4+ T-cell count and/or HIV viral load [14]. Furthermore, syphilis may have a more fulminant course in HIV-infected subjects, and progress more rapidly to neurosyphilis [13,16].

An HIV patient with ocular syphilis exhibited functional improvement and resolution of ocular inflammation after a specific antibiotic treatment. Sequelae included sectorial atrophy of the optic nerve with visual field loss and abnormalities of the retinal pigment epithelium [16].

## Conclusions

Our patient showed a probable *Treponema pallidum* reinfection . The definition of reinfection in syphilis is difficult, and both past and current research has tried to define it in relation to the introduction of new laboratory tests. Re-infection has been observed in HIV-infected patients with good immune-virological response [14]. Sexual behavior and patient follow-up remain the keys to identifying stage of infection [17].

Consequently, we wish to reiterate that unexplained external and internal ocular lesions such as eyelid lesions in HIV-infected patients should always raise the suspicion of syphilis. In this group, oral sex may be practiced as a form of “safer sex,” where the risk of HIV transmission was traditionally thought to be lower [18].

Epidemiological data indicates a worldwide reemergence of syphilis and a high degree of suspicion is necessary in view of its multitude of presenting ocular signs without pathognomonic features. Awareness of the reemergence of syphilis as well as a high degree of clinical suspicion can allow ophthalmologists to diagnose and treat the disease early, having a reasonably good visual prognosis following treatment with antibiotics.

### Consent

Written informed consent was obtained from the patient for publication of this case report and any accompanying images. A copy of the written consent is available for review by the Series Editor of this journal.

## Competing interests

The authors declare that they have no competing interests.

## Authors' contributions

All authors contributed to this work. SC and PDC carried out the literature search and drafted the manuscript; FDP, MT and VLV were involved in the direct clinical care (diagnosis, decision making, and treatment) of the reported patient. All authors involved in the preparation of the manuscript. All authors read and approved the final version of the manuscript.

## Pre-publication history

The pre-publication history for this paper can be accessed here:

http://www.biomedcentral.com/1471-2334/12/226/prepub
